# Peptide OPTX-1 From *Ornithodoros papillipes* Tick Inhibits the pS273R Protease of African Swine Fever Virus

**DOI:** 10.3389/fmicb.2021.778309

**Published:** 2021-12-03

**Authors:** Jingjing Wang, Mengyao Ji, Bingqian Yuan, Anna Luo, Zhenyuan Jiang, Tengyu Zhu, Yang Liu, Peter Muiruri Kamau, Lin Jin, Ren Lai

**Affiliations:** ^1^School of Life Sciences, University of Science and Technology of China, Hefei, China; ^2^Key Laboratory of Animal Models and Human Disease Mechanisms of Chinese Academy of Sciences/Key Laboratory of Bioactive Peptides of Yunnan Province, KIZ-CUHK Joint Laboratory of Bioresources and Molecular Research in Common Diseases, National Resource Center for Non-Human Primates, Kunming Primate Research Center, National Research Facility for Phenotypic & Genetic Analysis of Model Animals (Primate Facility), Sino-African Joint Research Center, and Engineering Laboratory of Peptides, Kunming Institute of Zoology, Chinese Academy of Sciences, Kunming, China; ^3^School of Life Sciences, Tianjin University, Tianjin, China; ^4^Kunming College of Life Science, University of Chinese Academy of Sciences, Kunming, China

**Keywords:** *Ornithodoros papillipes*, African swine fever virus, soft ticks, pS273R protease, vector

## Abstract

African swine fever virus (ASFV) is a large double-stranded DNA virus and causes high mortality in swine. ASFV can be transmitted by biological vectors, including soft ticks in genus *Ornithodoros* but not hard ticks. However, the underlying mechanisms evolved in the vectorial capacity of soft ticks are not well-understood. Here, we found that a defensin-like peptide toxin OPTX-1 identified from *Ornithodoros papillipes* inhibits the enzyme activity of the ASFV pS273R protease with a *K_i_*=0.821±0.526μM and shows inhibitory activity on the replication of ASFV. The analogs of OPTX-1 from hard ticks show more inhibitory efficient on pS273R protease. Considering that ticks are blood-sucking animals, we tested the effects of OPTX-1 and its analogs on the coagulation system. At last, top 3D structures represented surface analyses of the binding sites of pS273R with different inhibitors that were obtained by molecular docking based on known structural information. In summary, our study provides evidence that different inhibitory efficiencies between soft tick-derived OPTX-1 and hard tick-derived defensin-like peptides may determine the vector and reservoir competence of ticks.

## Introduction

African swine fever virus (ASFV), which causes a highly contagious and hemorrhagic disease of swine with a 100% mortality rate, is the only known DNA arbovirus and can be transmitted by *Ornithodoros* soft ticks (Kleiboeker et al., 1998). As a vector, soft ticks have ability to acquire and support replication of ASFV (Burrage, 2013; Ribeiro et al., 2015; Pereira de Oliveira et al., 2019). The viral titer and persistence were tested on the soft tick and virus combination, which highlights the vector and reservoir competence of *Ornithodoros* ticks for ASFV (Pereira De Oliveira et al., 2020). However, it was reported that even though viral DNA can be detected in many hard ticks, hard ticks are unlikely to be vectors of ASFV given the lack of virus replication (de Carvalho Ferreira et al., 2014). So far, the underlying mechanisms evolved in the contrasting vectorial capacity between hard ticks and soft ticks are not well-understood. Thus, it is essential to further investigate the interaction between ASFV and ticks.

Defensins are important host defense peptides (HDPs) found in vertebrates, invertebrates, and plants. They are important endogenous antimicrobial factors to combat invading pathogens and have therapeutic potential for infectious disease (Rothan et al., 2014; Boto et al., 2018; Bruzzoni-Giovanelli et al., 2018; Zhao et al., 2018). For example, a defensin-like antiviral peptide BmKDfsin4 from the scorpion *Mesobuthus martensii* Karsch has been reported to inhibit hepatitis B virus replication (Zeng et al., 2016). An1a, an antiviral peptide from the venom of the *Alopecosa nagpag* spider, has shown potent inhibitory activities targeting the NS2B–NS3 protease of DENV2 and ZIKV (Ji et al., 2019). Several antiviral peptides from ticks have also been identified (Talactac et al., 2017).

As a large DNA virus, ASFV capsid comprises 8,280 major capsid protein p72 and 60 penton protein copies (Liu et al., 2019). It is known that some polyprotein precursors cleaved by viral proteinases can yield structural proteins. pS273R is a specific SUMO-1 cysteine protease that catalyzes the maturation of the pp220 and pp62 polyprotein precursors into core–shell proteins (Li et al., 2020a). The proteolytic processes are important for the core maturation and infectivity of ASFV (Alejo et al., 2003). The approaches in describing the 3D structure of pS273R protease are very helpful in the development of anti-viral agents against ASFV (Li et al., 2020a; Liu et al., 2021). Moreover, in this study, the reported high-resolution structural basis of ASFV and pS273R is useful in understanding the vectorial capacity between hard ticks and soft ticks. Herein, we have found that the peptide toxin OPTX-1 identified from *Ornithodoros papillipes* inhibits the enzyme activity of the pS273R protease with a *K_i_*=0.821±0.526μM. Interestingly, we also found that the analogs of OPTX-1 from hard ticks show more inhibitory efficiency on pS273R protease. The molecular docking results show that OPTX-1 interacts with pS273R mainly through the active site in the core domain. Given the lack of effective treatments against ASFV, our results may also provide insights into anti-ASFV drugs development from natural origin.

## Materials and Methods

### Cell Lines and Viruses

PAM cells were prepared from a healthy 2-month-old pig (Huang et al., 2020). Lungs were removed from euthanized pigs, and then the bronchoalveolar lavage was performed. The collected PAMs were washed and cultivated in RPMI-1640 supplemented with 10% FBS, 0.1mM MEM non-essential amino acids, 1mM sodium pyruvate, 100U/ml penicillin, and 100μg/ml streptomycin in 5% CO_2_ at 37°C.

Vero cells (African green monkey kidney epithelial cell line) were obtained from Kunming Cell Bank, Kunming Institute of Zoology, Chinese Academy of Science. Vero cells were cultured in DMEM medium (Gibco, Waltham, MA, USA) supplemented with 10% fetal bovine serum (FBS), 100U/ml penicillin, and 100μg/ml streptomycin in 5% CO_2_ at 37°C.

### Protein Production

The codon-optimized cDNA of ASFV pS273R protease was synthesized (TsingKe Biotech, Co., Ltd., Beijing, China) as previously reported (Li et al., 2020a). The full-length pS273R protease was cloned into the pET-28b (Novagen) vector, and the sequence accuracy was verified. The recombinant plasmid of ASFV pS273R protease was transformed into *Escherichia coli* strain BL21(DE3) (TransGen Biotech, Beijing, China) and overexpressed. Protein expression was induced by 0.5mM IPTG (isopropyl-β-d-thiogalactopyranoside) and further purified on a Ni-NTA column. SDS-PAGE analysis revealed >98% purity of the final purified recombinant protein.

### Peptide Synthesis and Refolding

Peptides were synthesized by GL Biochem (Shanghai) Ltd. (Shanghai, China) and analyzed by reversed-phase high-performance liquid chromatography (RP-HPLC) and mass spectrometry to confirm their purity greater than 98%. The linear reduced peptide was dissolved in 0.1M Tris·HCl and 0.1M sodium chloride buffer (pH 8.0) at a final concentration of 30μM glutathione containing 0.1mM reduced glutathione and 0.5mM oxidized glutathione at 25°C for 24h. Oxidized and folded peptides were fractionated by analytical C_18_ RP-HPLC using a linear acetonitrile gradient, and the purity was detected by MALDI-TOF-MS (Luo et al., 2018).

### Protease Inhibition Assay

To test if the peptides inhibited pS273R protease activity, assays were conducted in 96-well black microplates utilizing Bz-Nle-Gly-Gly-Arg-AMC (GL Biochem Ltd., Shanghai, China) as a substrate (Andres et al., 2001). Monitoring was initiated, and the fluorescence of each well was recorded every 30s using an excitation of 360nm and emission of 460nm on a FlexStation microplate reader (Molecular Devices, Sunnyvale, CA, USA). Results were determined as relative fluorescence units (RFU). Curves were generated using the GraphPad Prism 6 software (Version 6.01, GraphPad Software, Inc., San Diego, CA, 2012, USA).

The effects of peptides on thrombin (T4393, Sigma-Aldrich, USA) were determined as described previously (Tang et al., 2020). Substrates for thrombin (β-Ala-Gly-Arg-pNA diacetate, T3068) were purchased from Sigma-Aldrich (USA). The rate of protease hydrolyzate was monitored continuously at 405nm from 20 to 60min. The inhibition constant *K_i_* was determined according to the reported methods.

### ASFV Infection and Quantification

For virus infection, PAM cells were infected with ASFV SY18 strain (GenBank: MH766894) at 0.1 MOI with or without OPTX-1 administration for 48h in a biosafety facility. The total DNA were extracted with TIANamp Genomic DNA Kits (DP304-03, Tiangen, Beijing, China). qRT-PCR was performed on the StepOnePlus Real-Time PCR Systems (Thermo Fisher Scientific, Waltham, MA, USA), and the primer sequences are p72-F (TTAGGTACTGTAACGCAGCA) and p72-R (ATGGCAT CAGGAGGAGC; Li et al., 2020b). Swine *Actb* gene (ACTB-F, ACCTTCTACAATGAGCTGCG and ACTB-R, CTGGATGGCTAC GTACATGG) was used as a reference gene for relative quantification.

### Blood Coagulation Assays

Blood coagulation assays were performed according to our previous study (Luan et al., 2017). Healthy human plasma was collected from the Kunming Blood Center, and blood was prepared by mixing a 1:9 volume of trisodium citrate (0.13M) and blood, with plasma then obtained by centrifugation at 3,000rpm for 20min at 4°C. Twenty microliter of platelet-poor plasma (PPP) collected from healthy human subjects was dispensed into round-bottomed 96-well plates, and then the testing sample which was dissolved in 80μl of HEPES buffer (with 0.15M NaCl, pH 7.4) was added to the plates. After incubation for 10min at room temperature, 50μl of 0.025M calcium chloride (CaCl_2_) was added to the plates, and the clotting time was recorded. Clotting time was calculated by measuring the time to half maximal increase in absorbance. All the analyses were monitored by a microplate spectrophotometer (BioTek Instrument, Inc., Winooski, VT, USA) at the absorbance of 650nm.

### Cell Viability Assay

Cell viability was evaluated by conventional 3-(4,5-dimethyl-2-thiazolyl)-2,5-diphenyl-2H-tetrazolium bromide (MTT) reduction assays in 96-well plates. After a 24h treatment by testing sample, MTT was added to each well to a final concentration of 0.5mg/ml and incubated at 37°C for 4h. The MTT solution was then removed, and dimethyl sulfoxide (DMSO) was added to solubilize the MTT-formazan crystals in living cells. The absorbance of the resulting solution was measured at 570nm.

### Molecular Docking

The Modeller v9.18 software was used to construct a model of three tick defensins, OPTX-1, persulcatusin, and longicin by homology modeling and then used ZDOCK v3.0.2 software to perform molecular docking (Pierce et al., 2014; Webb and Sali, 2021).

### Statistical Analysis

Figures were generated using the GraphPad Prism 6 software (Version 6.01, GraphPad Software, Inc., San Diego, CA, 2012, USA). Data are given as mean±SEM. Statistical analysis was performed using two-tailed Student *t* test. Value of *p*≤0.05 was considered significant.

## Results

### Refolding of OPTX-1 and Hard Tick-Derived Defensins

**Figure 1:**
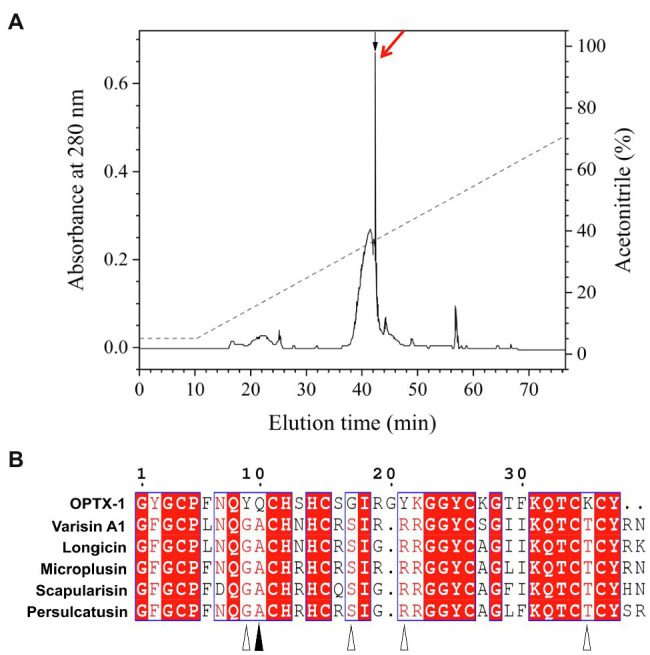
Refolding of OPTX-1 and the sequence alignment with its analogs. **(A)** OPTX-1 folded into the correct structure was collected by analysis chromatography, which was purified using a RP-HPLC column (Unisil C18 column, 5μm particle size and 10×250mm). Elution was performed at a flow rate of 1.5ml/min with the indicated gradients of acetonitrile in 0.1% (v/v) trifluoroacetic acid (TFA) in water. **(B)** Similarity of OPTX-1 to hard tick-derived defensins. The relevant sequence GenBank accession numbers are: OPTX-1: FJ222575.1, Varisin A1: AY181027.1, longicin: EU035973.1, Microplusin: MK818522.1, Scapularisin: AY660970.1, Persulcatusin: AB469201.1.

Synthesized peptides were oxidized and folded before use. As shown in Figure 1A and Supplementary Figure 1, peptides were fractionated by analytical C_18_ RP-HPLC using a linear acetonitrile gradient, and the purity was further confirmed by MALDI-TOF-MS (Supplementary Figure 2). The observed molecular mass of refolded OPTX-1 was 4166.6Da by using a positive ion and linear mode. Considering that the sequence contains six cysteine residues, which likely form three intra-molecular disulfide bridges, it is corresponded with the predicted molecular mass. As shown in Figure 1B, OPTX-1 exhibited both sequence identities and diversities of other known hard tick-derived defensin-like peptides.

### OPTX-1 Inhibits the Activity of pS273R and the Replication of ASFV

**Figure 2:**
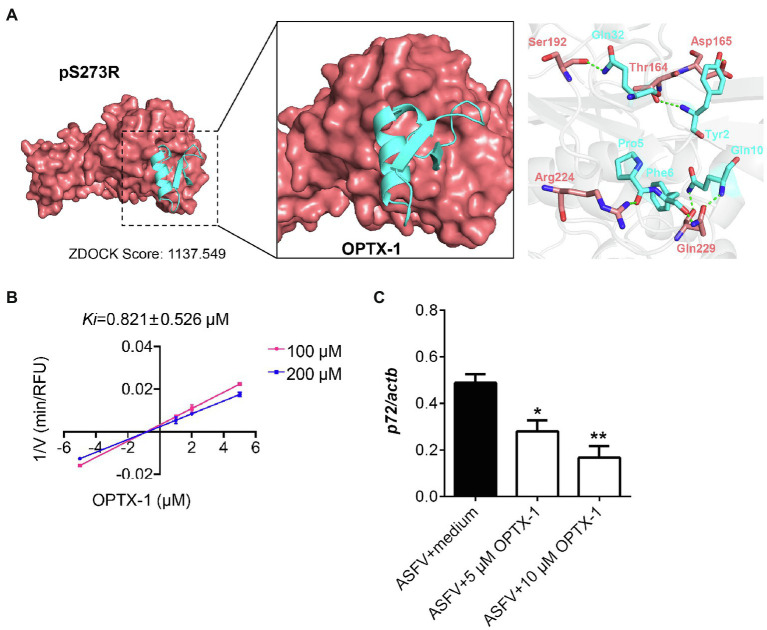
OPTX-1 inhibits the activity of pS273R and the replication of ASFV. **(A)** Molecular docking between OPTX-1 and pS273R. The residues are shown as stick models. The hydrogen bonds are shown as dashed green lines. **(B)** The Lineweaver–Burk plot shows that OPTX-1 is a competitive inhibitor of the pS273R protease, and the *K_i_* value was determined by the method of Dixon. V is the reaction rate. **(C)** OPTX-1 inhibits the replication of ASFV in PAM cells at 48hpi. Data represent three independent experiments in **A,B** and two independent experiments in **C**. *p<0.05 and **p<0.01.

The pS273R protease is essential for ASFV replication and maturation. It has been regarded as an important antiviral target (Li et al., 2020a). The docking model of the peptides–pS273R complex indicated that the active site of pS273R core domain may directly interact with OPTX-1 and other hard tick-derived defensins by forming several hydrogen bonds and/or hydrophobic bonds (Figure 2A and Supplementary Figure 3A). The Lineweaver–Burk plot shows that OPTX-1 is a competitive inhibitor of the pS273R protease, and the *K_i_* value was determined as 0.821±0.526μM by the method of Dixon (Figure 2B). The hard tick-derived defensins were also demonstrated to be competitive inhibitors of the pS273R protease with much more remarkable inhibitory effect (Supplementary Figure 3B). To determine the anti-ASFV activity of OPTX-1, we analyzed the replication of ASFV by qRT-PCR at 48-h post-infection. In line with the observations, we found that OPTX-1 inhibited ASFV infection in PAM cells at the concentration 5μM and 10μM (Figure 2C). The results indicated that OPTX-1 is a competitive inhibitor of pS273R protease of ASFV by interacting with its active site and hence inhibits the replication of ASFV.

### OPTX-1 Shows Inhibitory Effects on Coagulation System

**Figure 3:**
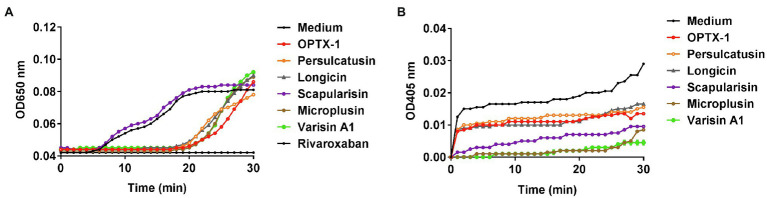
OPTX-1 shows inhibitory effects on coagulation system. **(A)** Effects of 10μM of tick-derived peptides on plasma recalcification time. Ten micrometer of Rivaroxaban was set as a positive control. **(B)** Real-time detection of the thrombin inhibitory effects of tick defensins at 10μM.

Ticks are obligate blood-feeding arthropod vectors and are responsible for highly prevalent tick-borne diseases (TBDs) worldwide. It is reasonable to speculate that the tick-derived peptides possess anticoagulant activity. As shown in Figure 3A, OPTX-1 and other hard tick-derived defensins show anticoagulant effects in plasma-based coagulation assays except scapularisin. Further investigation demonstrated that the activity of thrombin was inhibited by OPTX-1 (Figure 3B). These data suggest that OPTX-1 and hard tick-derived defensins may be conducive to blood feeding of ticks by inhibiting the host coagulation system.

### OPTX-1 Has Relative Low Cytotoxicity

**Figure 4:**
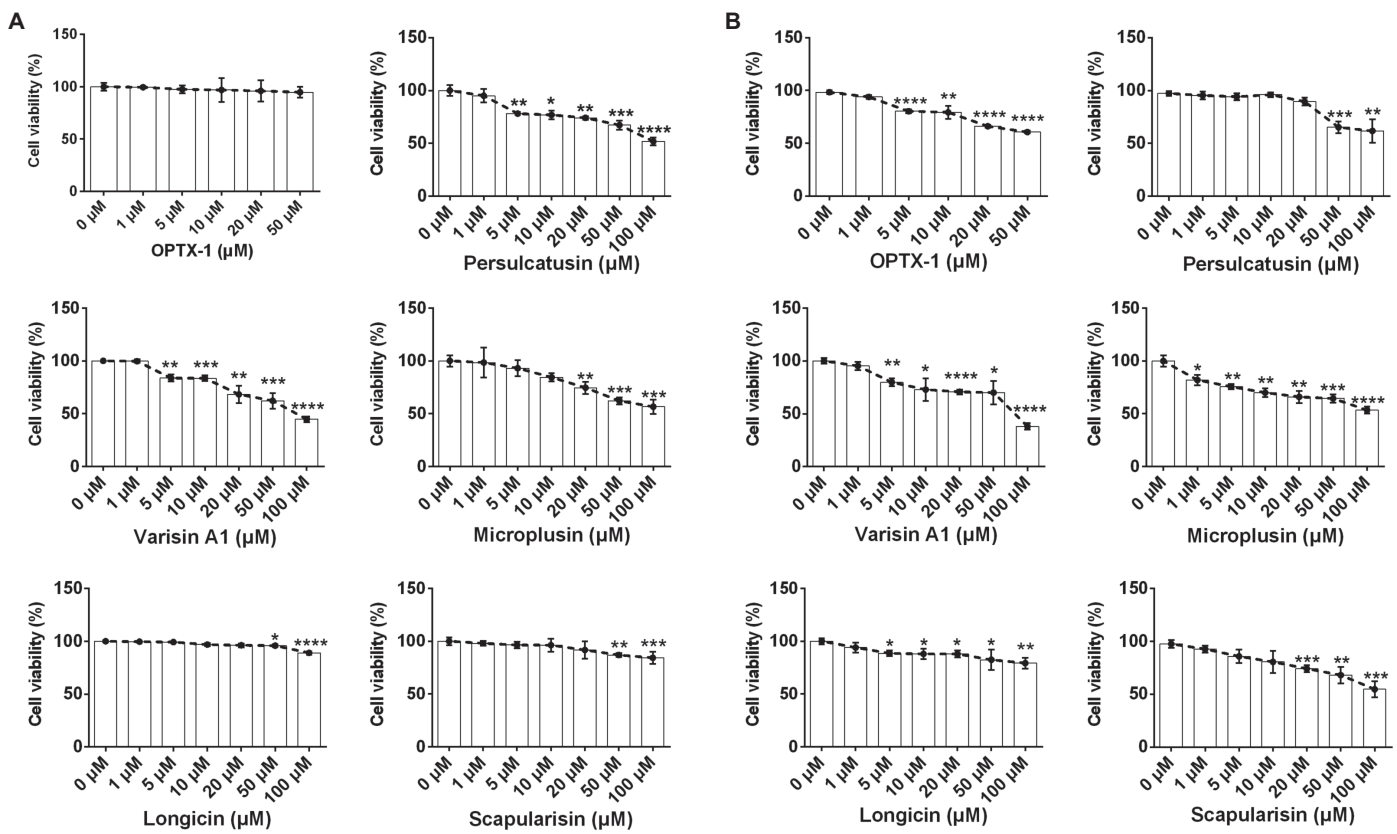
OPTX-1 has relative low cytotoxicity. **(A)** The cytotoxicity of OPTX-1 and its analogs on Vero cells. **(B)** The cytotoxicity of OPTX-1 and its analogs on PAM cells. Data represent three independent experiments and are presented as mean±SEM. ^*^p<0.05; ^**^p<0.01; ^***^p<0.001; and ^****^p<0.0001.

ASF currently has no effective pharmacological treatment. As competitive inhibitors of pS273R protease of ASFV, OPTX-1 and its analogs are expected to provide peptide precursor molecules for the development of therapeutic drugs for ASF, for further evaluation, cytotoxicity was assessed by using the MTT test. As shown in Figure 4A, OPTX-1 showed less cytotoxicity on Vero cells than other defensins. The cytotoxicity of OPTX-1 on PAM cell was more significant than on Vero cells (Figure 4B). Taken together, these data suggest that OPTX-1 as well as hard tick-derived defensins has relative low cytotoxicities and might be good template for anti-ASF drug design.

## Discussion

ASFV affects domestic and wild members of the Suidae family, leading to a wide range of symptoms from chronic or persistent infection to acute hemorrhagic fever, and inflicts up to 100% mortality (Gallardo et al., 2015). The main routes for disease transmission are direct contact between susceptible and sick animals or their fluids or excretions, and indirect contact through contaminated feed, pork meat, people, vehicles, or fomites (Mur et al., 2012; Dee et al., 2019). Until now, there are no effective drugs and vaccines for ASF. In 2018, the ASFV has been spread to China and other Asian countries. Other than the direct contact transmission, another possible way of the spread of ASFV is vectorial transmissions by some species of *Ornithodoros* soft ticks. We assumed that the vectorial efficiencies of ticks are depended on the successful but limited replication of ASFV.

As a crucial SUMO-1-specific protease of ASFV, pS273R represents an attractive therapeutic target (Li et al., 2020a). This study implicated that tick defensins which inhibit the pS273R protease of ASFV may play an important role in vector borne transmission. The cleavage of polyproteins by viral encoded proteases is a common strategy (Yost and Marcotrigiano, 2013). The polyprotein precursors pp220 and pp62 expressed by ASFV have cleavage sites of the consensus sequence Gly-Gly-Xaa, which can be processed by the pS273R (Alejo et al., 2003). In this study, we have found that OPTX-1 is a competitive inhibitor of pS273R protease of ASFV and inhibits the replication of ASFV *in vitro*.

The docking model indicated that the active site of pS273R core domain directly interacts with the peptides mainly through the hydrogen bonds. The cysteine protease activity of pS273R can be abolished by mutation of the predicted catalytic histidine and cysteine residues or inhibited by sulfhydryl-blocking reagents (Andres et al., 2001; Alejo et al., 2003). However, the sequence similarly of the defensins included in this study indicates that some amino acid residues are distinguished markedly between soft tick (Tyr9, Gln10, Gly17, Tyr21, and Lys35) and hard tick (Gly9, Ala10, Ser17, Arg21, and Thr35) origin. In particular, the Gln10 of OPTX-1 is the estimated key sites which may form hydrogen bonds with Gln229 of pS273R. The Lineweaver–Burk plot shows that tick defensins are competitive inhibitors of the pS273R protease. As we all know, ticks are blood-feeding arthropods. Tick-derived peptides may possess anticoagulant activity. We found that OPTX-1 and other hard tick-derived defensins show anticoagulant and thrombin inhibitory effects except scapularisin. These findings indicate that the effects of tick defensins on pS273R protease and coagulant system are relatively independent. Thus, further investigations are needed to fully elucidate the underlying biological functions and mechanisms of tick defensin-like peptides.

Defensins are known major immunoregulatory components of ticks that have been shown to provide protection against gram-negative and gram-positive bacteria, fungi, viruses and protozoan parasites (Taute et al., 2015; Wang et al., 2015; Yada et al., 2018). Functional mature defensins are cationic peptides with molecular mass up to 4kDa, containing six cysteine residues that form characteristic intra-molecular disulfide bridges (Chrudimska et al., 2010). Considering that the ASFV assembly process is strictly depended on the correct spatial and temporal maturation of the pp220 and pp62 polyproteins, tick defensins may limit the replication of ASFV by inhibiting the pS273R. Inhibitors of pS273R protease of ASFV are expected to be potential therapeutic drugs for ASF. As OPTX-1 and hard tick-derived defensins have relative low cytotoxicities, they might provide valuable structural information for further anti-ASF small molecular inhibitor drug development targeting pS273R. Previous experimental studies showed that some species of *Ornithodoros* soft ticks can be orally infected, maintained and transmitted ASFV vertically (transstadially and transovarially) among ticks, and horizontally to naive pigs. Several studies emphasized that vector competence was not only related to the tick but also to the ASFV strain. Thus, it is necessary to test the inhibitory effects on pS273R protease as well as the virus replications of tick defensins with different ASFV variants.

## Data Availability Statement

The raw data supporting the conclusions of this article will be made available by the authors, without undue reservation.

## Author Contributions

JW, MJ, BY, AL, ZJ, TZ, and YL conducted the experiments. RL and LJ designed the experiments and provided guidance for the research. LJ analyzed the data and wrote the paper. PK revised the manuscript. All authors contributed to the article and approved the submitted version.

## Funding

This work was supported by the African Swine Fever Research Emergency program of the Chinese Academy of Sciences (CAS) (KJZD-SW-L06-02). LJ is partly supported by the National Natural Science Foundation of China (NSFC) grant (31900331 and 32070444), Science and Technology Department of Yunnan Province (202001AW070019), Chinese Academy of Sciences “Light of West China” program and Youth Innovation Promotion Association (2019378). RL is partly supported by the NSFC grant (3193001521761142002), the CAS grants (XDB31000000, SAJC202103, KFJ-PTXM-28SAJC201606, and KGFZD-135-17-011), and Yunnan Province grant (2019-YT-053, 202002AA 100007, and 2019ZF003).

## Conflict of Interest

The authors declare that the research was conducted in the absence of any commercial or financial relationships that could be construed as a potential conflict of interest.

## Publisher’s Note

All claims expressed in this article are solely those of the authors and do not necessarily represent those of their affiliated organizations, or those of the publisher, the editors and the reviewers. Any product that may be evaluated in this article, or claim that may be made by its manufacturer, is not guaranteed or endorsed by the publisher.
