# Supplementary material for: Peptide OPTX-1 From Ornithodoros papillipes Tick Inhibits the pS273R Protease of African Swine Fever Virus
Source: Front Microbiol. 2021 Dec 3;12:778309. doi: 10.3389/fmicb.2021.778309 (PMC8678048; doi:10.3389/fmicb.2021.778309)
Supplement: Supplementary file 1 [file Data_Sheet_1.PDF]

Supplementary Material

1 Supplementary Figures

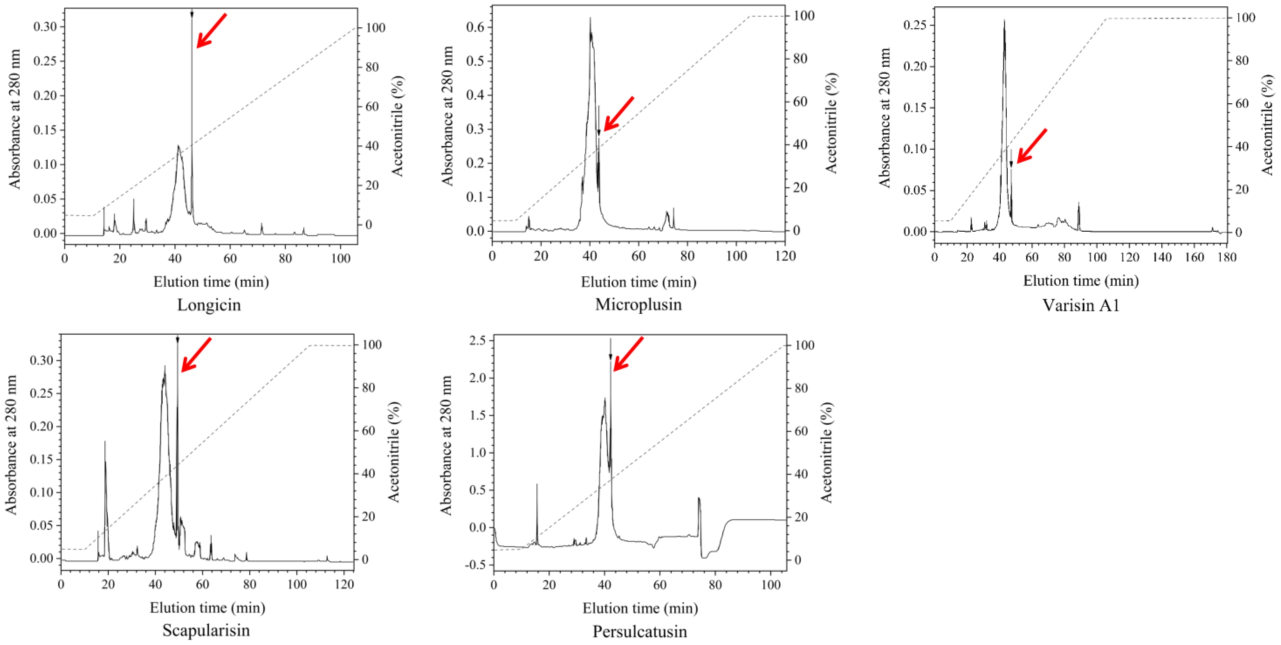

Supplementary Figure 1. Refolding of the hard tick-derived defensins.

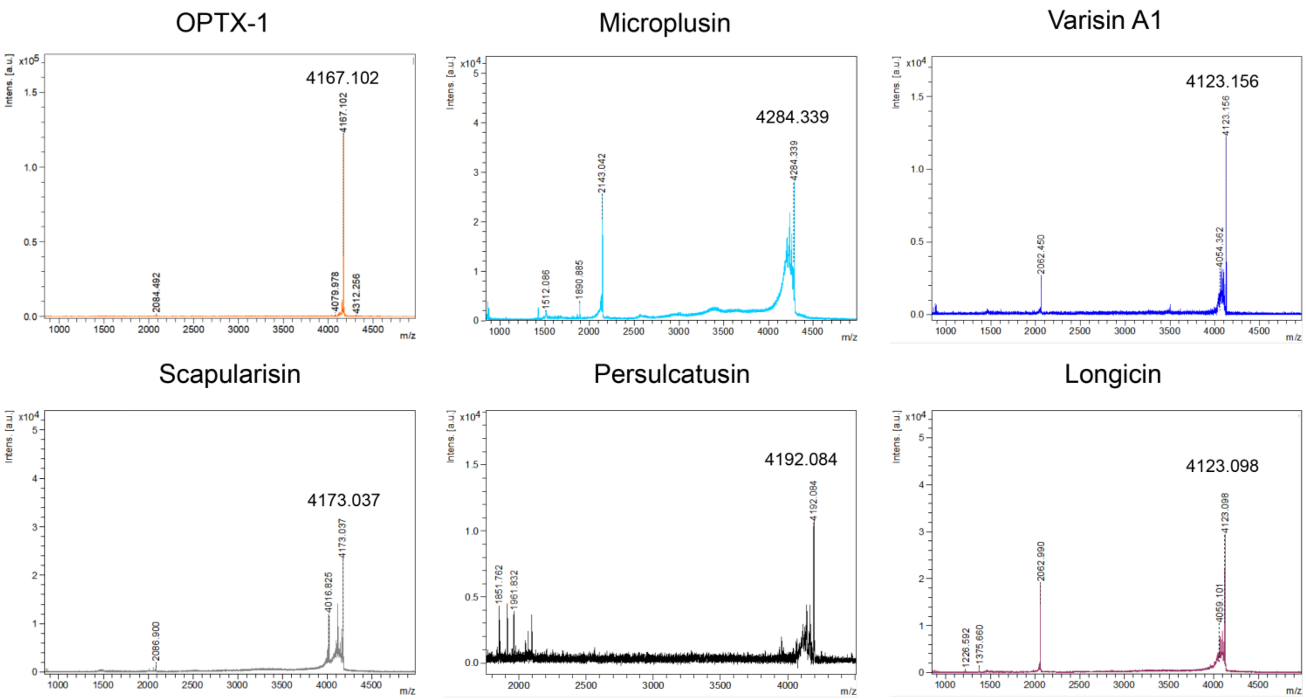

Supplementary Figure 2. The average molecular weights of refolded OPTX-1 and the analogs were determined by using MALDI-TOF with the LP model.

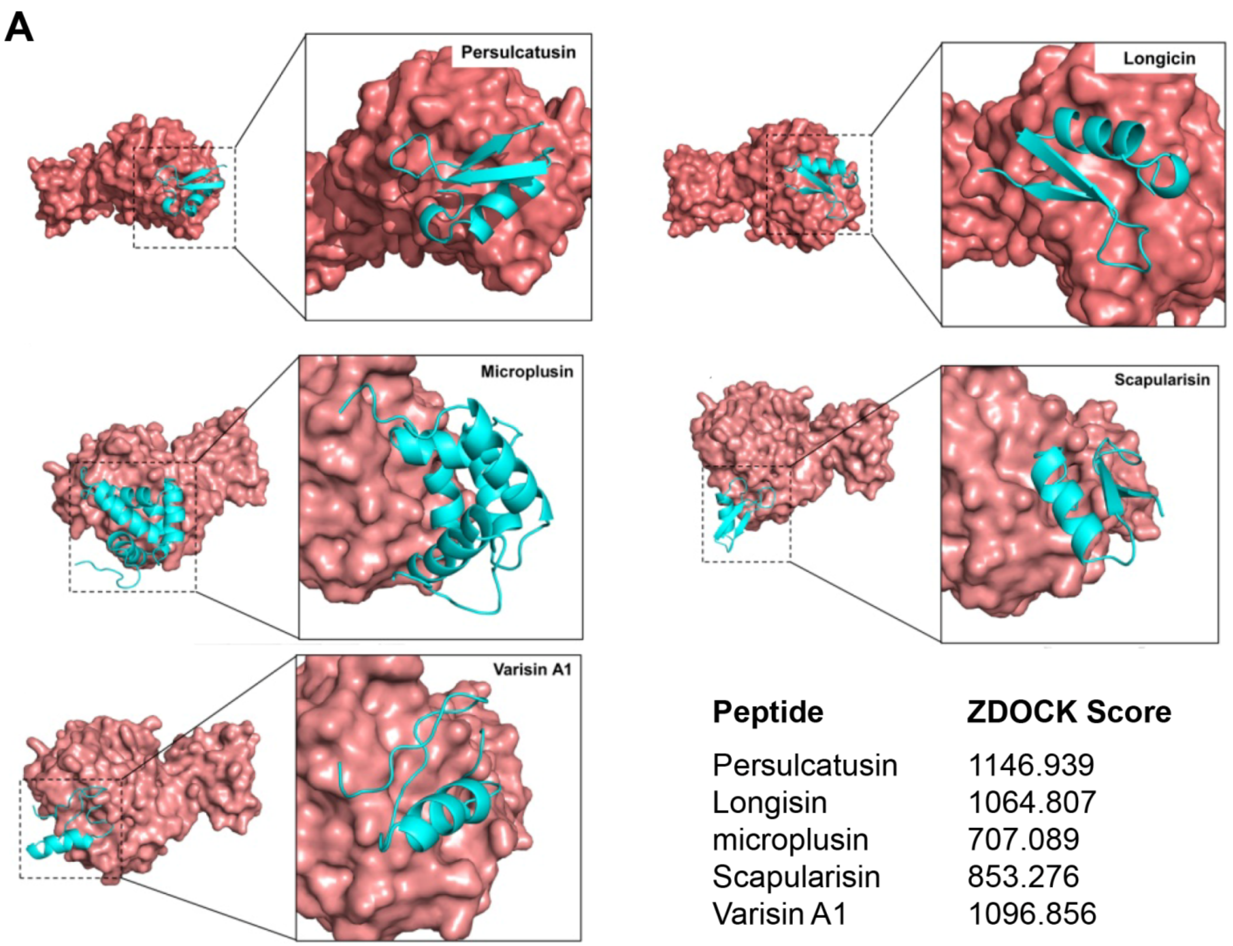

Peptide	ZDOCK Score
Persulcatusin	1146.939
Longisin	1064.807
microplusin	707.089
Scapularisin	853.276
Varisin A1	1096.856

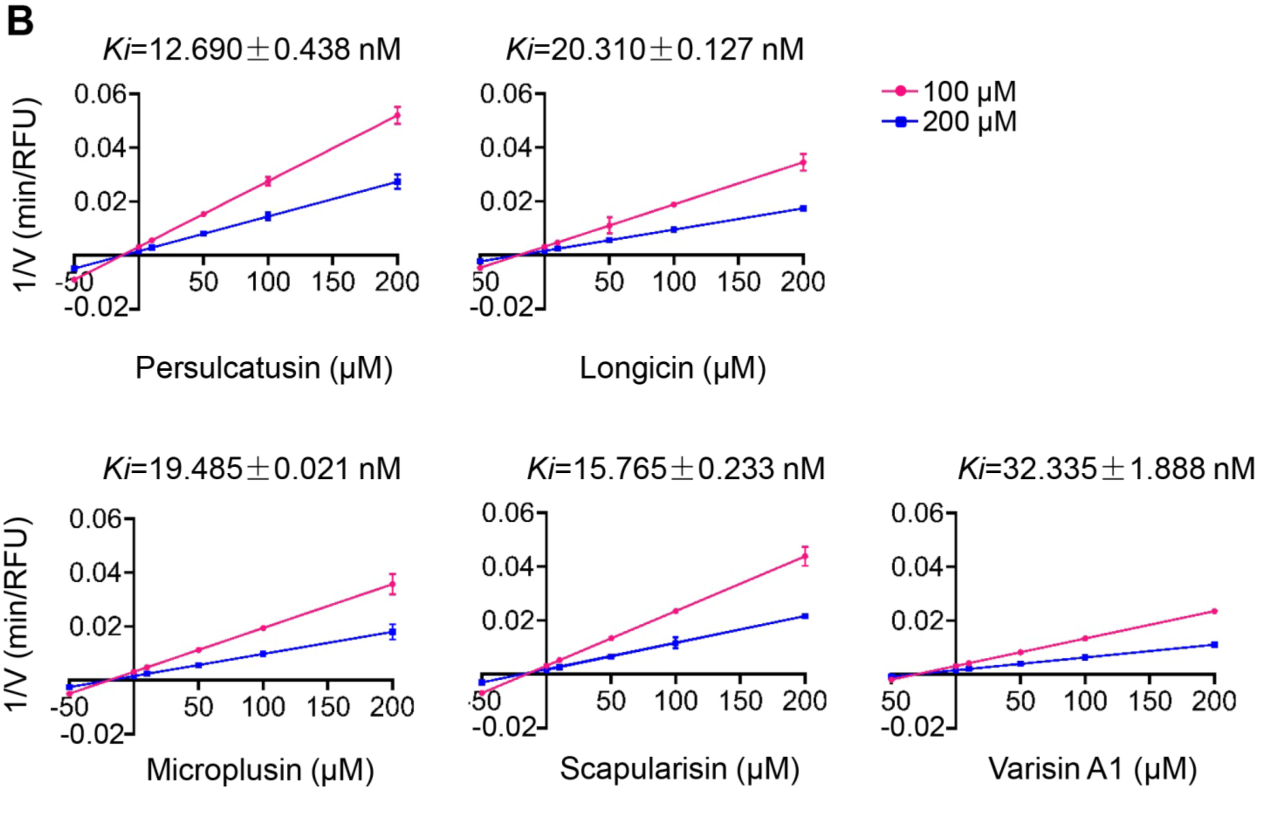

Supplementary Figure 3. Hard tick defensins inhibits the pS273R of ASFV (related to Figure 2). (A) Molecular docking between defensins and pS273R. (B) The Lineweaver–Burk plot shows that hard tick defensins are competitive inhibitors of the pS273R protease, and the *Ki* value was determined by the method of Dixon. V is the reaction rate.
